# GPR30 disrupts the balance of GABAergic and glutamatergic transmission in the spinal cord driving to the development of bone cancer pain

**DOI:** 10.18632/oncotarget.11867

**Published:** 2016-09-06

**Authors:** Jie Luo, Xiaoxia Huang, Yali Li, Yang Li, Xueqin Xu, Yan Gao, Ruoshi Shi, Wanjun Yao, Juying Liu, Changbin Ke

**Affiliations:** ^1^ Institute of Anesthesiology & Pain (IAP), PET-CT, Institute of Anesthesiology and Department of Anesthesiology, Taihe Hospital, Hubei University of Medicine, Shiyan City, 442000, Hubei Province, China; ^2^ Department of Nephrology, Taihe Hospital, Hubei University of Medicine, Shiyan City, 442000, Hubei Province, China

**Keywords:** bone cancer pain, spinal cord, GPR30, excitatory transmission, inhibitory transmission

## Abstract

Cancer induced bone pain is a very complicated clinical pain states that has proven difficult to be treated effectively due to poorly understand of underlying mechanism, but bone cancer pain (BCP) seems to be enhanced by a state of spinal sensitization. In the present study, we showed that carcinoma tibia implantation induced notable pain sensitization and up-regulation of G-protein-coupled estrogen receptor (GPR30) in the spinal cord of rats which was reversed by GPR30 knockdown. Further studies indicated that upregulation of GPR30 induced by cancer implantation resulted in a select loss of γ-aminobutyric acid-ergic (GABAergic) neurons and functionally diminished the inhibitory transmission due to reduce expression of the vesicular GABA transporter (VGAT). GPR30 contributed to spinal cord disinhibition by diminishing the inhibitory transmission via upregulation of α1 subunit and downregulation of γ2 subunits. GPR30 also facilitated excitatory transmission by promoting functional up-regulation of the calcium/calmodulin-dependent protein kinase II α (CaMKII α) in glutamatergic neurons and increasing the clustering of the glutamate receptor subunit 1 (GluR1) subunit to excitatory synapse.

Taken together, GPR30 contributed to the development of BCP by both facilitating excitatory transmission and inhibiting inhibitory transmission in the spinal cord. Our findings provide the new spinal disinhibition and sensitivity mechanisms underlying the development of bone cancer pain.

## INTRODUCTION

Cancer induced bone pain is a very complicated clinical pain states that has proven difficult to be treated effectively [1–3]. The mechanism responsible for bone cancer pain (BCP) is poorly understood, but BCP seems to be enhanced by a state of spinal sensitization [4, 5]. The development of central sensitization results in mild noxious sensory stimuli being perceived as highly noxious stimuli (hyperalgesia), and normally non-noxious sensory stimuli being perceived as noxious stimuli (allodynia) [4]. This procession is regulated by a balance between excitatory and inhibitory signaling transmission in the spinal cord [6]. Disturbing the balance of excitatory-inhibitory transmission through increase excitatory or decrease inhibitory transmitter in the spinal cord would increase neuronal excitability [7] and enhance pain perception. Thus, we hypothesized that imbalance of excitatory-inhibitory transmission mediated by γ-aminobutyric acid-ergic (GABAergic) and glutamatergic neurons in the spinal cord would result in the development of BCP.

Spinal cord network circuitry consists of excitatory and inhibitory neurons [8]. The net output of the spinal cord represents a balance between spinal excitatory processes and inhibitory interneurons. Any abnormal changes, such as increasing the excitability of excitatory neurons, selective loss of GABAergic interneurons, decreased expression level of GABA or GABA_A_ receptors in the spinal cord may result in neuronal hypersensitivity or “disinhibition” and therefore increase the spinal excitability [9, 10]. Emerging data has suggested that both increasing the excitatory transmission and reduction in tonic and phasic inhibitory control of inhibitory interneurons in the spinal cord are dominantly responsible for the amplification of pain perception [11, 12].

Several recent studies suggest that G-protein-coupled estrogen receptor (GPR30) is a functional membrane estrogen receptor involved in estrogen signaling. However, this function is not universally accepted. A little study showed that GPR30 related the sex relative behavior in female mice, such as Lordosis [13] and endometriosis [14]. In fact, recent studies mainly focus on the physiological role of GPR30 in the nervous system rather than in reproduction systems.

GPR30 has been shown to be localized at the whole nervous system, from cortex to the spinal cord and dorsal root ganglion [15–17]. The existing data overwhelmingly demonstrates the potential role of GPR30 in regulating neuronal activity related to pain [14, 15, 18–20]. Other studies have proposed that GPR30 modified the balance between GABAergic and glutamatergic transmission in the basolateral nucleus of the amygdala of ovariectomized-stressed mice [21]. We hypothesized that GPR30 contributed to BCP and the underling mechanism dues to diminish GABAergic interneurons by selectively losing of GABAergic neurons and regulation of GABA_A_ receptors and enhance the Glutamatergic transmission in the spinal cord.

## RESULTS

### Upregulation of GPR30 in the ipsilateral dorsal horn of cancer bearing rats

**Figure 1:**
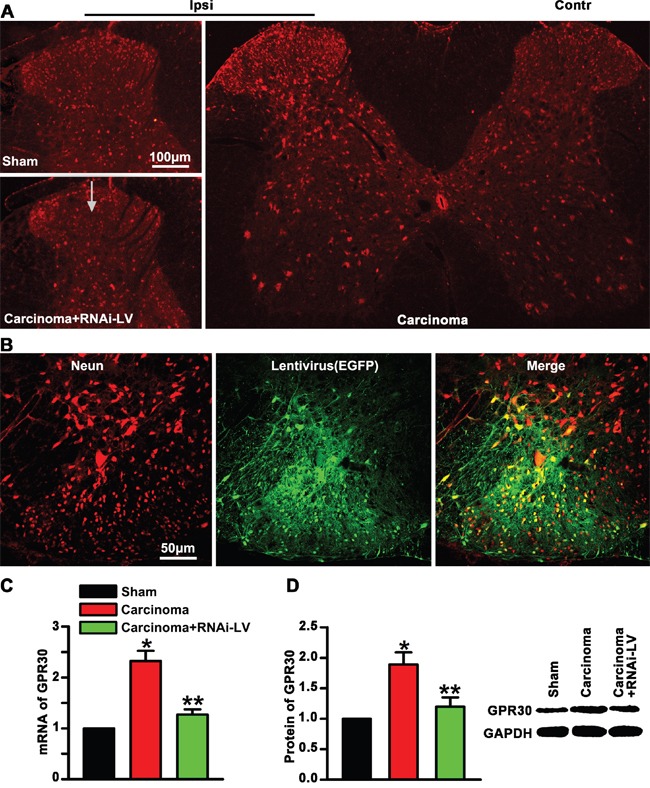
Upregulation of GPR30 in the spinal cord of cancer-bearing rats **A.** Immunofluorescent labeling using an antibodies against GPR30(red) in the spinal cord of sham, cancer-bearing, and RNAi-LV treatment rats. Scale bar 100μm, a white arrow marked the location of lentivirus injection. The spinal cord stain showed that GPR30 was increase in the ipsilateral dorsal horn of cancer-bearing rats. **B.** Immunofluorescent labeling of neuron with an antibody of anti-Neun (red) to show the effect of lentivirus (green) transfection. Scale bar 50μm. **C, D.** Quantitative analysis of GPR30 by RT-PCR and western blot in the spinal cord of sham, carcinoma, RNAi-LV treatment rats. A blot sample was showed on the right. Values represent the mean±SEM, n=4. *P<0.05 compared with the sham; **P<0.05 compared with the carcinoma.

To investigate whether GPR30 was involved in the development of BCP, firstly, the expression of GPR30 in lumbar (L4-L6) spinal dorsal horn was examined using immunofluorescent labeling with an anti-GPR30. Immunofluorescent staining of transverse sections of spinal cord showed that GPR30 was localized in the spinal cord and increased in ipsilateral dorsal horn of cancer-bearing rats which was reversed by spinal cord injection of RNAi-LV, (Figure 1A). To knock down GPR30 in carcinoma inoculation rats, we injected a recombinant RNAi lentivirus (RNAi-LV) targeting GPR30 into the ipsilateral dorsal horn. The virus drove the expression of enhanced green fluorescent protein (EGFP) to allow the detection of the transfected tissue. A recombinant lentivirus without targeting sequence was used as negative control (NC-LV, data not shown). Immunofluorescent labeling of spinal cord slices with an antibody of anti-Neun showed that most of the neurons in the dorsal horn were transfected by lentivirus, (Figure 1B). Quantitative analysis of GPR30 expression using RT-PCR and Western blot respectively indicated that carcinoma inoculation induced upregulation of GPR30 in the spinal cord which were reversed significantly by RNAi-LV, but not NC-LV(data not shown), (Figure 1C, 1D). The results suggested that GPR30 was probably a candidate molecular involving the development of BCP.

### GPR30 contributed to the development of BCP

**Figure 2:**
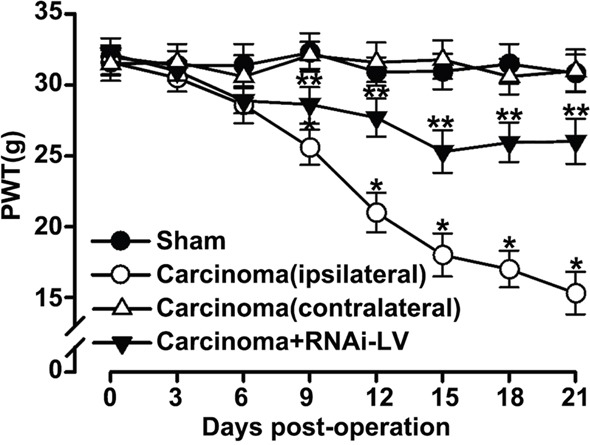
GPR30 induced the development of BCP Mechanical allodynia was measure with paw withdrawal threshold (PWT) in sham, cancer-bearing, and RNAi-LV treatment rats. Note that the carcinoma implantation induced decrease of PWT on the ipsilateral paw, but not on the contralateral side which was reversed by RNAi-LV treatment. Values represent the mean±SEM, n=6. *P<0.05 compared with the sham; **P<0.05 compared with the carcinoma (ipsilateral).

To further evaluate whether GPR30 was essential for the development of BCP, a local injection of carcinoma cells directly into rat right tibia bone cavity was used to mimic clinical BCP and mechanical allodynia was measured with paw withdraw threshold (PWT). The RNAi-LV was injected into the ipsilateral dorsal horn to knock down GPR30 expression. Before cancer cells or lentivirus injection, the overall mean baseline PWT to noxious stimuli was similar in all of rats. We had shown that carcinoma inoculation resulted in significant bone cancer-related pain behaviors on the ipsilateral paw, but not on the contralateral side which was reversed by GPR30 knockdown (Figure 2). These results indicated that GPR30 was essential for the development of BCP.

### GPR30 functionally inhibited the GABAergic interneurons

**Figure 3:**
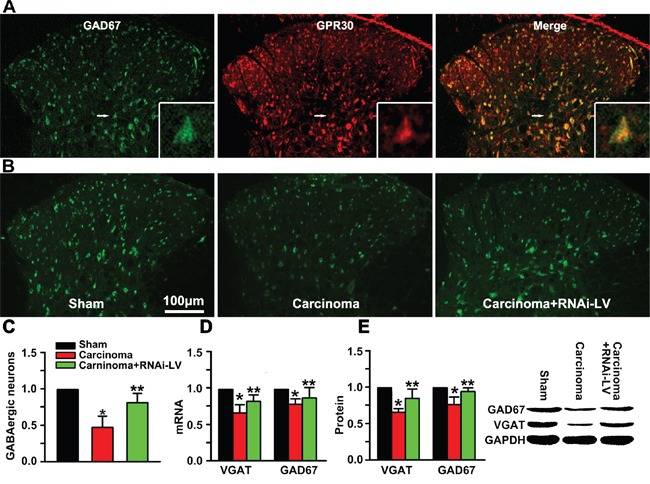
GPR30 diminished inhibitory transmission of GABAergic neurons **A.** Immunofluorescent labeling using antibodies against GPR30(red) and GABAergic interneuron(green) in the spinal cord of sham, cancer-bearing, and RNAi-LV treatment rats. Scale bar 100μm. The white rectangle outlined the highly magnified cell showing the co-localization of GPR30 and GAD67. **B.** Immunofluorescent labeling using antibody against GAD67 of sham, cancer-bearing, and RNAi-LV treatment rats **C.** Quantitative analysis of GABAergic neurons in the spinal cord of sham, carcinoma, carcinoma+siGPR30-LV rats. Values represent the mean±SEM, n=6 in each group. *P<0.01 compared with the sham; ^**^P<0.01 compared with the carcinoma. **D, E.** Quantitative analysis of GAD67 and VGAT by RT-PCR and Western blot respectively in rat spinal cord of sham, carcinoma, carcinoma+siGPR30-LV rats. A blot sample was showed on the right. Values represent the mean±SEM, n=5. *P<0.05 compared with the sham; **P<0.05 compared with the carcinoma.

Pain procession seems to be dynamically and powerfully controlled by GABAergic neurons in the spinal cord. To investigate whether GPR30 diminished the function of GABAergic interneurons during the development of BCP, RNAi-LV targeting GPR30 was injected into the ispilateral dorsal horn of cancer-bearing rats, the localization of GPR30 on GABAergic neurons was analyzed using inmmunofluorescent double staining with antibodies of GPR30 and GAD67 (a marker of GABAergic interneuron). The merged images showed that GPR30 expressed in GABAergic interneurons (Figure 3A). Quantitative analysis of the images showed that the number of GABAergic neurons decreased in cancer implantation rats indicating great loss of the GABAergic neurons. However down-regulation of GPR30 prevented the loss of GABAergic neurons (Figure 3B, 3C).

The function of GABAergic neurons relies on the accumulation of GABA into synaptic vesicles which was controlled by the vesicular GABA transporter (VGAT).

To further investigate whether GPR30 inhibited the function of GABAergic neurons via VGAT, quantitative analysis of GAD67 and VGAT was performed with RT-PCR and western blot respectively. Both the mRNA and protein of GAD67 and VGAT decreased in carcinoma inoculation rats which were reversed by GPR30 knockdown (Figure 3D, 3E). The results indicated that GPR30 diminished inhibitory transmission of GABAergic neurons by inducing a selective loss of GABAergic neurons and decreasing the transport of GABA through inhibition of VGAT.

### GPR30 mediated spinal disinhibition by regulating different GABA_A_ receptor subunits

**Figure 4:**
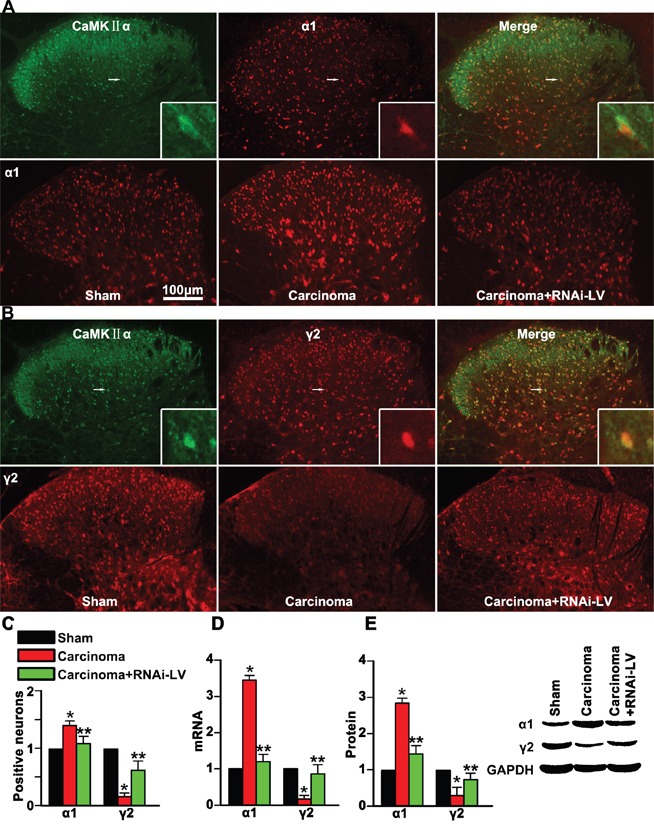
GPR30 diminished GABAergic inhibitory on excitatory neuron **A.** Double immunofluorescent labeling using antibodies against α1 (red) and CaMKII α (green) in the spinal cord of sham, cancer-bearing, and RNAi-LV treatment rats. **B.** Double immunofluorescent labeling using antibodies against γ2 (red) and CaMKII α(green) in the spinal cord of sham, cancer-bearing, and RNAi-LV treatment rats. Scale bar 100μm. The highly magnified images showed that both α1 and γ2 expressed in the spinal glutamatergic neurons. **C.** Quantification of α1 and γ2 positive cells in the dorsal horn of sham, carcinoma, RNAi-LV treatment rats. Values represent the mean±SEM, n=6 in each group. *P<0.05 compared with the sham; ^**^P<0.05 compared with the carcinoma. **D, E.** Quantitative analysis of α1 and γ2 subunits using RT-PCR and Western blot respectively of sham, carcinoma and RNAi-LV treatment rats, a blot sample showed on the right. Values represent the mean±SEM, n=5 in each group. *P<0.01 compared with the sham; ^**^P<0.01 compared with the carcinoma.

Nociceptive transmission of excitatory neurons is modulated by postsynaptic inhibition of GABA receptors in the dorsal horn. However, the function of GABA_A_ receptors was regulated by the expression of different GABA_A_ receptor subunits, such as α1 and γ2 subunits. To explore whether GPR30 regulated the inhibitory modulation of GABAergic neurons on the excitatory neurons and induced spinal disinhibition in the spinal dorsal horn via α1 and γ2 subunits, we used RNAi-LV to knock down GPR30 in the dorsal horn and examined the expression of α1 or γ2 subunits on the excitatory neurons (marked with the calcium/calmodulin-dependent protein kinase IIα, CaMKII α) using double immunofluoresence staining. The merge pictures showed that both α1 and γ2 were localized on CaMKII α positive neurons (Figure 4A, 4B). Quantitative analysis indicated that carcinoma implantation induced a significant increase of α1 and decrease of γ2 positive cells. A local injection of RNAi-LV targeting GPR30 into the dorsal horn reversed the changes of α1 and γ2 induced by carcinoma implantation, (Figure 4C). The results were further confirmed by quantitative analysis using RT-PCR and western blot respectively, (Figure 4D, 4E). These results indicated that GPR30 may diminish the inhibitory transmission of GABAergic neurons by upregulation of α1 subunit and downregulation of γ2 subunits.

### GPR30 promoted functional up-regulation of CaMKII α in glutamatergic neurons

**Figure 5:**
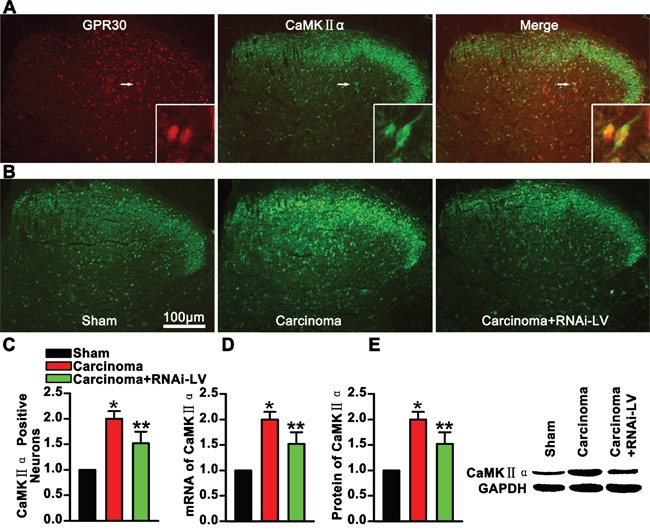
GPR30 enhanced excitatory transmission of glutamatergic neurons **A.** Double immunofluorescent labeling using antibodies against GPR30(red) and CaMKII α (green) in the spinal cord, The white rectangle outlined the highly magnified cells (marked by white arrow) showing the co-localization of GPR30 and CaMKII α. **B.** Immunofluorescent labeling using antibodies against CaMKII α (green) in the spinal cord of sham, cancer-bearing, and RNAi-LV treatment rats. Scale bar 100μm. **C.** Quantification of CaMKII α positive neurons of sham, carcinoma and RNAi-LV treatment rats. Values represent the mean±SEM, n=6, *P<0.05 compared with the sham; ^**^P<0.05 compared with the carcinoma. **D, E.** Quantitative analysis of CaMKII α by RT-PCR and Western blot in rat spinal cord of sham, carcinoma, RNAi-LV treatment rats. A blot sample showed on the right. Values represent the mean±SEM, n=5. *P<0.05 compared with the sham; ^**^P<0.05 compared with the carcinoma.

CaMKII induced delivery of tagged a-amino-3-hydroxy-5-methyl-4-isoxazole propionic acid (AMPA) receptors into synapses that controlled and executed activity-dependent synaptic plasticity involving in the development of chronic pain. To demonstrate whether the GPR30 enhanced the function of glutamatergic neurons, antibodies against CaMKII α and GPR30 were used in double immunofluorescence staining of spinal cord slices. The images showed that CaMKII α was located in the dorsal horn and the magnified images showed that GPR30 was localized in the CaMKII α positive neurons, (Figure 5A). Quantitative analysis of magnified images showed a significant increase of CaMKII α positive neuron in cancer implantation rats which was reversed by knockdown of GPR30, (Figure 5B, 5C). Further quantitative analysis the expression of CaMKII α was performed using RT-PCR and western blot respectively. Both the mRNA and protein of CaMKII α increased in carcinoma inoculation rats which were reversed by GPR30 knockdown, (Figure 5D, 5E). These results implied that GPR30 functionally enhanced the excitatory transmission of glutamatergic neurons in the spinal cord of cancer-bearing rats.

### GPR30 facilitated excitatory transmission in the dorsal horn

**Figure 6:**
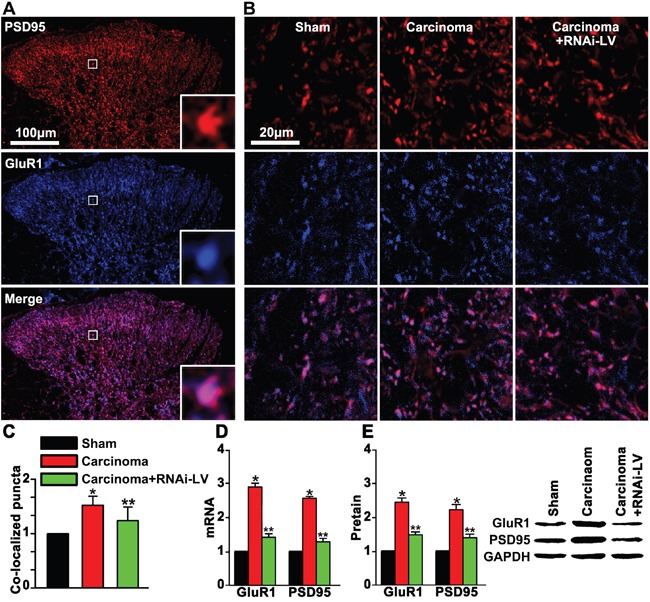
GPR30 up-regulated GluR1 and PSD95 in the dorsal horn of cancer-bearing rats **A.** Double staining of PSD95 (red) and GluR1 (blue) in the dorsal horn of sham, carcinoma and RNAi-LV treatment rats. The white square region was zoomed in (bottom-right) to show the co-localization of GluR1and PSD95. Scale bar 100μm. **B.** Highly magnified picture of GluR1and PSD95 puncta. Scale bar 20μm. **C.** Quantitative analysis of co-localization puncta of GluR1 and PSD95. **D, E.** Quantitative analysis of of GluR1 and PSD95 by RT-PCR and Western blot respectively, a blot sample showed on the right. Values represent the mean±SEM, n=5, *P<0.05 compared with the sham; ^**^P<0.05 compared with the carcinoma.

GluR1, a major subunit of AMPA receptors, was involved in pain-related hyperexcitability [23, 24]. To explore whether GPR30 facilitated excitatory transmission by increasing the clustering of GluR1 subunit to the excitatory synapse. RNAi-LV was used to knock down GPR30 and double staining of GluR1 and PSD-95 (a key postsynaptic scaffolding protein of excitatory synaptic transmission) was used to show the postsynaptic clustering of GluR1 on the excitatory neurons. We showed that carcinoma implantation significantly increased the expression of GluR1 and PSD-95 in the dorsal horn of cancer bearing rats which were remarkably reversed by GPR30 knockdown, (Figure 6A, 6B). Quantitative analysis of double staining pictures indicated that co-localized puncta of GluR1 and PSD-95 were increased significantly in cancer bearing rats and reversed by GPR30 knockdown, (Figure 6C). The expression of GluR1 and PSD-95 were further confirmed by RT-PCR and western blot respectively, (Figure 6D, 6E). Taken together, these results indicated that up-regulation of GPR30 promoted excitatory transmission of glutamatergic neurons in the spinal cord of carcinoma-implanted rats.

## DISCUSSION

The present study provides spinal sensitivity evidence for the contribution of GPR30 to the development of BCP. Upregulation of GPR30 in cancer-bearing rats induced selective loss of GABAergic neurons and diminishment of GABAergic inhibition on excitatory neurons. GPR30 also promoted cluster of AMPA receptors on postsynaptic membrane that facilitated excitatory transmission. Our findings provide a new spinal sensitivity mechanism underlying the development of bone cancer pain.

Inhibitory neurotransmission in the spinal dorsal horn is at the foundation of the gate control theory of pain and has been the subject of intense investigation. Thus, inhibition of GABAergic neurons, decrease of inhibitatory transmitter release or blocking those receptors would result disinhibition and induce persistent pain [25]. Here, we showed now unequivocal evidence that inhibition in the spinal dorsal horn is pathologically reduced during the development of BCP.

Loss of inhibitory function of GABA neurons in particular may be the main mechanism by which abnormal neural hyperactivity occurs, leading to exaggerated sensory processing during the development of chronic pain. In the present study, we showed a direct evidence that GPR30 induced a selective loss of GABAergic neurons in the dorsal horn during the development of BCP. These results illustrated a potential new role of GPR30 involving in the development of BCP.

GABA is inhibitory neurotransmitters in the spinal dorsal horn. [26]. Diminished GABA release will reduce both presynaptic (influencing synaptic inflow) and postsynaptic (modulating dorsal horn neuron excitability) GABA_A_ receptors-mediated inhibition. So, loading of GABA into synaptic vesicles via the VGAT is an essential step in inhibitory neurotransmission. As a result of the evidence linking alterations in GABAergic neurotransmission to various pain disorders. In the present study, upregulation of GPR30 induced by carcinoma implantation functionally inhibited the expression VGAT. These results indicated that GPR30 decreasing the release of GABA by modulation of VGAT, and therefore diminished the inhibitory neurotransmission, increasing the neural hyperactivity, leading to exaggerated sensory processing and contributing to BCP.

Postsynaptic GABA_A_ receptors-mediated inhibition is functionally regulated by the subunits of GABA_A_ receptors. These receptors are consist of diverse α and β subunits together with the γ2 subunit. Approximately half of GABA_A_ receptors contain α1 subunits. Plasticity in α1 subunit expression is associated with changes in CNS excitability [27]. Previous study reported that α1 subunit deletion resulted in reduction of GABA transporter, and therefore increased extracellular GABA concentration resulting in“tonic” inhibition, a powerful modulator of neuronal excitability [28]. The γ2 subunit of GABA_A_ receptors chloride channels is required for normal channel function and for postsynaptic clustering of these receptors GABA_A_ receptors. Loss of GABA_A_ receptor clusters in mice deficient in the γ2 subunit is paralleled by loss of synaptic GABAergic function [29, 30]. In the present study, we showed that sarcoma implantation induced upregulation of α1 subunit and downregulation of γ2 on the excitatory neurons, which was reversed by GPR30 knockdown. These results suggested that GPR30 contributed to the development of BCP through inhibition of GABAergic neurotransmission. The functional inhibition on GABAergic neurons was probably by decrease of extracellular GABA concentration and diminishment of postsynaptic clustering of GABA_A_ receptors via regulating α1 and γ2 GABA_A_ receptors subunits.

Thus, we concisely illustrated a spinal disinhibitory mechanism mediated by GPR30 involving in the development of BCP and provide a potential therapeutic opportunities to alleviate BCP by restoring normal inhibition of spinal cord.

The spinal sensitization, exaggerating perception of pain stimulus is mediated by excitatory glutamatergic synaptic transmission that results an increased excitability of nociceptive neurons in the spinal cord. Excitatory synaptic transmission is critically dependent on maintaining an optimal number of postsynaptic AMPA subtype of ionotropic glutamate receptors (GluRs) [23, 24, 31]. The CaMKIIα induces delivery of tagged GluRs into synapses that controls and executes synaptic plasticity involving in the painful procession.

In the present, we showed that sarcoma tibia implantation induced upregualtion of GPR30 as well as increasing CaMKIIα and GluR1 in the excitatory neurons. Further study showed that GluR1 postsynaptically co-located with PSD95. GPR30 knockdown reversed cancer induced-bone pain and decreased the expression CaMKIIα and GluR1, and therefore reduced the excitatory synapses. These results indicated that GPR30 facilitated excitatory transmission, promoted spinal sensitization and contributed to BCP through CaMKIIα-dependent postsynaptic regulation of GluR1. Thus, inhibition of the excitatory transmission by downregulation of GPR30 was probably an optional therapeutic strategy to alleviate BCP.

The present study showed that GPR30 regulated functionally both on the excitatory and inhibitory transmission in the spinal cord and contributed to cancer induced bone pain. These effects of GPR30 on the pain regulation were probably duo to mediating some of the rapid signal transduction events, such as calcium mobilization [32]. However, calcium was the important physiological regulator for the function of glutamatergic and GABAergic neurons [33–37], which may illustrate the physiological basis and provide an approach to insight into the diversity function of GPR30 on the nervous system.

Overall, this study illustrated GPR30-mediated mechanism underline the development of BCP through changing the balance of excitatory and inhibitory transmission in the spinal cord and provided a new insight for BCP management, which means a consideration to both directed at preventing a loss of endogenous inhibitory control systems and diminishing the excitatory transmission in the spinal cord.

## MATERIALS AND METHODS

### Animals

All experimental procedures and protocols used in this study were reviewed and approved in advance by the Animal Care and Use Committee of Hubei University of Medicine. And also this study was adhered to the guidelines of the Committee for Research and Ethical Issues of IASP published in PAIN®, 16 (1983) 109-110. Experiments were carried out on female Sprague Dawley rats, provided by the Institute of Laboratory Animal Science, Hubei University of Medicine. The rats were kept under controlled conditions (24±0.5°C, 12h alternating light-dark cycle, food and water *ad libitum*).

### Preparation of Walker 256 carcinoma cells

Walker 256 rat mammary gland carcinoma cells (0.5 ml, 2×10^7^ cells/ml) were injected into the abdominal cavity of a Sprague Dawley rat. After 6 - 7 days, cells were harvested from 5 ml ascitic fluid of above rat. Cells were counted with a hemocytometer, then diluted to achieve a final concentration (1×10^6^ cells/ml) for injection and maintained on ice prior to surgery. For the sham group, Walker 256 cells were prepared in the same final concentrations for injection and boiled for 20 min.

### BCP model

A rat model was established on female Sprague Dawley rats using Walker 256 rat mammary gland carcinoma cells as previously described [22]. In briefly, animals were anesthetized with isoflurane (3% induction, 2% maintenance), then superficial incision was made in the skin overlying the right patella after disinfected with 75% v/v ethanol. The tibia was carefully exposed and a 23-gauge needle was inserted into the intramedullary canal of the bone. It was then removed and replaced with a long thin blunt needle attached to a 10μl syringe containing carcinoma cells. A volume of 10μl containing Walker 256 cells (10^4^ cells), or boiled cells was injected into the bone cavity. Following injection, the entry site on the bone was sealed with bone wax and the skin was closed. After surgery, rats were placed on a warm pad for recovery and then in separated cages under conditions as mentioned above.

### Behavioral studies

Throughout the experiment, examiners were blind to the treatment allocation of each animal. Pain-related behaviors were tested before carcinoma injections and then tested on days3, 6, 9, 12, 15, 18, and 21 after carcinoma injection. Rats were allowed to habituate for a period of 30 min, Mechanical allodynia was assessed using a dynamic plantar esthesiometer (UgoBasile, Stoelting, IL, USA). Animals were placed in enclosures on an elevated wire mesh and responses to punctate mechanical stimulation were assessed using the esthesiometer. A straight metal filament (0.5 mm diameter) was orientated beneath the pad until it touched the plantar surface of the hind paw and began to exert an upward force. The force required to elicit a withdrawal response (paw withdrawal threshold, PWT) was measured in grams when the paw was withdrawn or the preset cut-off was reached (50 g). Five values were taken alternately on both ipsilateral (operated side) and contralateral hind paws at intervals of 15 s.

### Lentivirus constructs and microinjection

Lentivirus constructs and microinjection were performed as our previously described [22]. We generated shRNAs, corresponding to siRNA sequences known to knock down GPR30. The shRNAs were synthesized as an inverse repeat separated by a loop for each sequence and inserted downstream of the U6 promoter in the lentiviral vector GV118. Lentiviruses were generated by triple transfection of ~80% confluent HEK293FT cells (Invitrogen). The same vector backbone but carrying the GFP protein was used as negative control. Lentiviruses were harvested in serum-free medium 3 days later, filtered and concentrated in primed Centricon Plus-20 filter devices (Millipore). Lentivirus vector construction and production were completed by Shanghai Gene Chem Co. Ltd., China. The viral titre of the viral stocks was 1.0×10^9^ TU/ml. Following carcinoma implantation, T13-L1 vertebrae were identified for laminectomy to uncover lumbar spinal cord at levels L4–L6. Using a glass capillary (30±5μm diameter), viral solution was injected into dorsal horn in two sites separated 2 mm along the spinal cord (2μl/site; flow rate of 0.4μl/min). The needle was positioned on the right at 0.4mm aside from the midline, and then it was deepened into the parenchyma to 0.5 mm below the piamater to reach the dorsal horn.
